# Surgical Management of Chronic Proximal Hamstring Avulsion Using Hamstring Allograft

**DOI:** 10.7759/cureus.93722

**Published:** 2025-10-02

**Authors:** Julio C Gali, Igor S de Novais, Leonardo A Carletti, Luiza G Gomes, Cláudio G Martins

**Affiliations:** 1 Surgery, Faculty of Medical Science and Health, Pontifical Catholic University of São Paulo, Sorocaba, BRA; 2 Radiology, Dr. Miguel Soeiro Hospital, Sorocaba, BRA

**Keywords:** allograft, avulsion, chronic, proximal hamstring tendon, surgical treatment

## Abstract

Chronic proximal hamstring avulsions are uncommon injuries that may result in significant functional impairment. Late presentation often includes reduced control of the affected limb during the eccentric phase of hamstring activation, particularly noticeable during activities such as running or brisk walking. Additional complaints may include posterior thigh cramping, subjective muscle weakness, sensations of instability or "giving way," and symptoms consistent with sciatica or radiculopathy. When significant tendon retraction is present, surgical reconstruction can be technically challenging due to factors such as tendon scarring, compromised tissue quality, and increased distance from the ischial tuberosity. In cases where primary repair is not feasible, hamstring allografts may be employed to bridge the defect and re-establish continuity of the musculotendinous unit.

## Introduction

Proximal hamstring avulsion is an uncommon injury, predominantly affecting individuals aged over 30 years old, with no clear gender predominance. Men are usually injured during sports, while women often sustain injuries during daily activities [1]. Bodendorfer et al., in a systematic review and meta-analysis, reported that the mean age in the chronic injury group was 39.3 years, with males comprising 68.5% of the cases [2]. Similarly, Bertiche et al., in a systematic review and meta-analysis, found that the average age of patients undergoing surgical treatment for proximal hamstring avulsion is approximately 40 years [3]. The primary mechanism typically involves forceful hip flexion with an extended knee, leading to complete avulsion of two or three tendons, most often the semitendinosus and biceps femoris [4,5].

Surgical treatment is recommended for patients with a complete three-tendon rupture or those with two-tendon tears exhibiting more than 2 cm of retraction [6]. Larson et al., on the other hand, consider surgery in cases of chronic complete proximal hamstring rupture when patients experience persistent weakness that limits daily activities, poor leg control during the eccentric phase of gait, recurrent spasms or cramping, and occasionally symptoms of sciatica [7].

Chronic cases, often defined as those treated beyond six to 12 weeks post injury, pose significant challenges due to fibrosis and tendon adherence to adjacent tissues, including the sciatic nerve [2,3,8,9]. Scarred and retracted tendons are difficult to mobilize, and neurolysis is often required when the sciatic nerve is encased in fibrotic tissue [10-12]. In chronic settings, greater knee flexion is frequently necessary during surgery to aid tendon approximation [12].

There is a higher incidence of neurological complications in chronic proximal hamstring repairs when compared to acute cases. Jokela et al. reported chronic symptoms caused by the pressure effect of scar tissue formation near the sciatic nerve [4]. Bodendorfer et al. observed that neurological complications occurred in 4.87% of chronic cases [2], while a broader cohort analysis by Joleka et al. revealed even higher rates, with neurologic complications present in 9.04% of chronic injuries versus 1.66% in acute ones [4]. Similarly, French et al. reported a higher incidence of neurological symptoms in chronic repairs (5.1%) compared to acute repairs (0.7%) [13].

We describe a surgical technique utilizing hamstring (semitendinosus and gracilis) allografts to bridge the gap and restore continuity of the musculotendinous unit. This approach enables anatomic reconstruction, promotes functional recovery, and may contribute to significant pain reduction. However, there is still no consensus on the optimal surgical approach for chronic proximal hamstring avulsions with significant retraction and nerve involvement. This report addresses this gap by describing a reproducible hamstring allograft technique aimed at restoring tendon continuity and function.

## Technical report

Surgical technique

**Figure 1:**
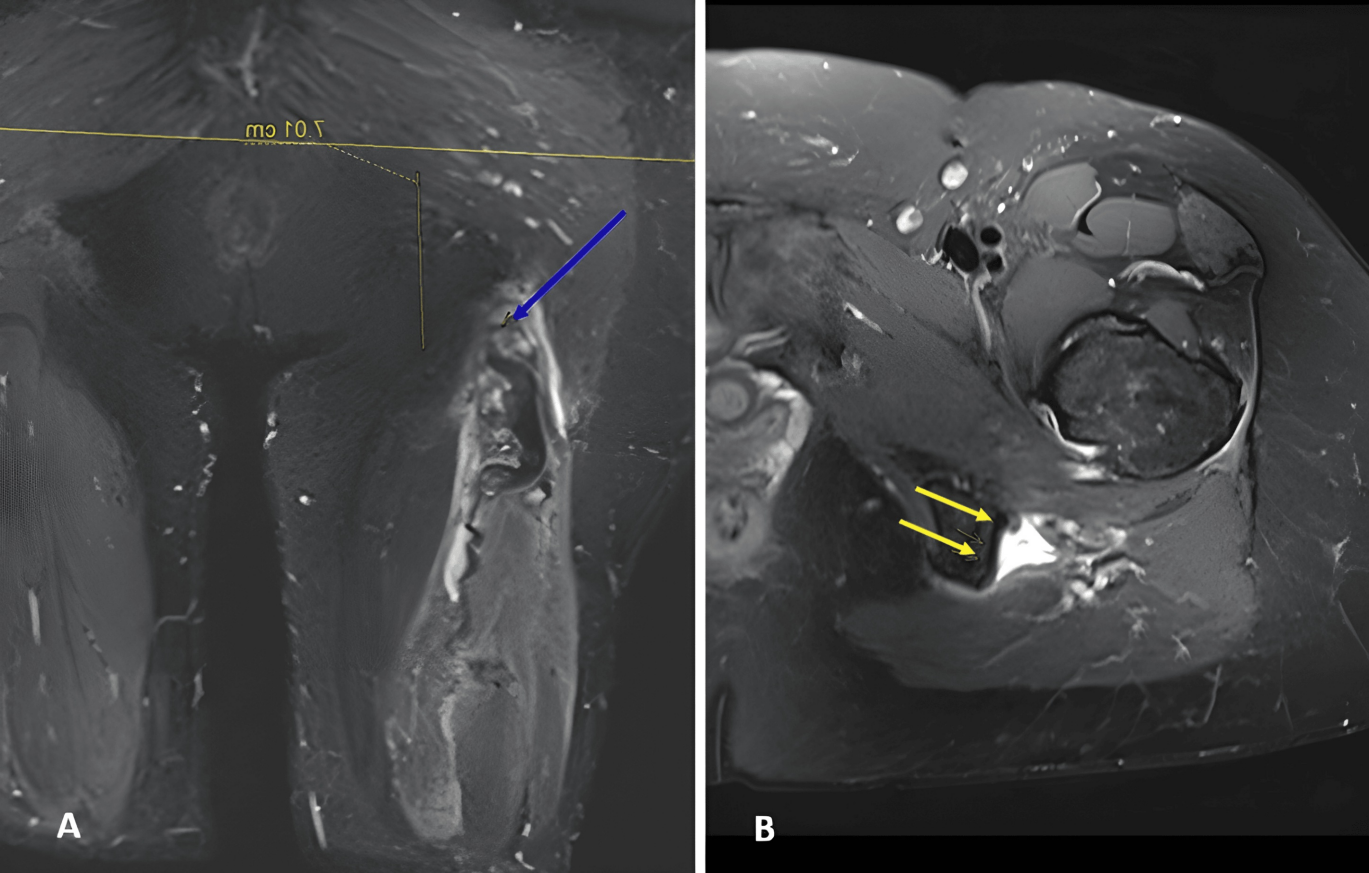
Preoperative MRI (A) Preoperative coronal STIR magnetic resonance image showing the retracted tendon stump and its distance from the ischial tuberosity (blue arrow); (B) Preoperative axial T2 fat-suppressed image demonstrating complete avulsion of the hamstring tendons (two yellow arrows). STIR: Short Tau Inversion Recovery Image Source: Faculty of Medical Science and Health, Pontifical Catholic University of São Paulo; Used with Permission

**Figure 2:**
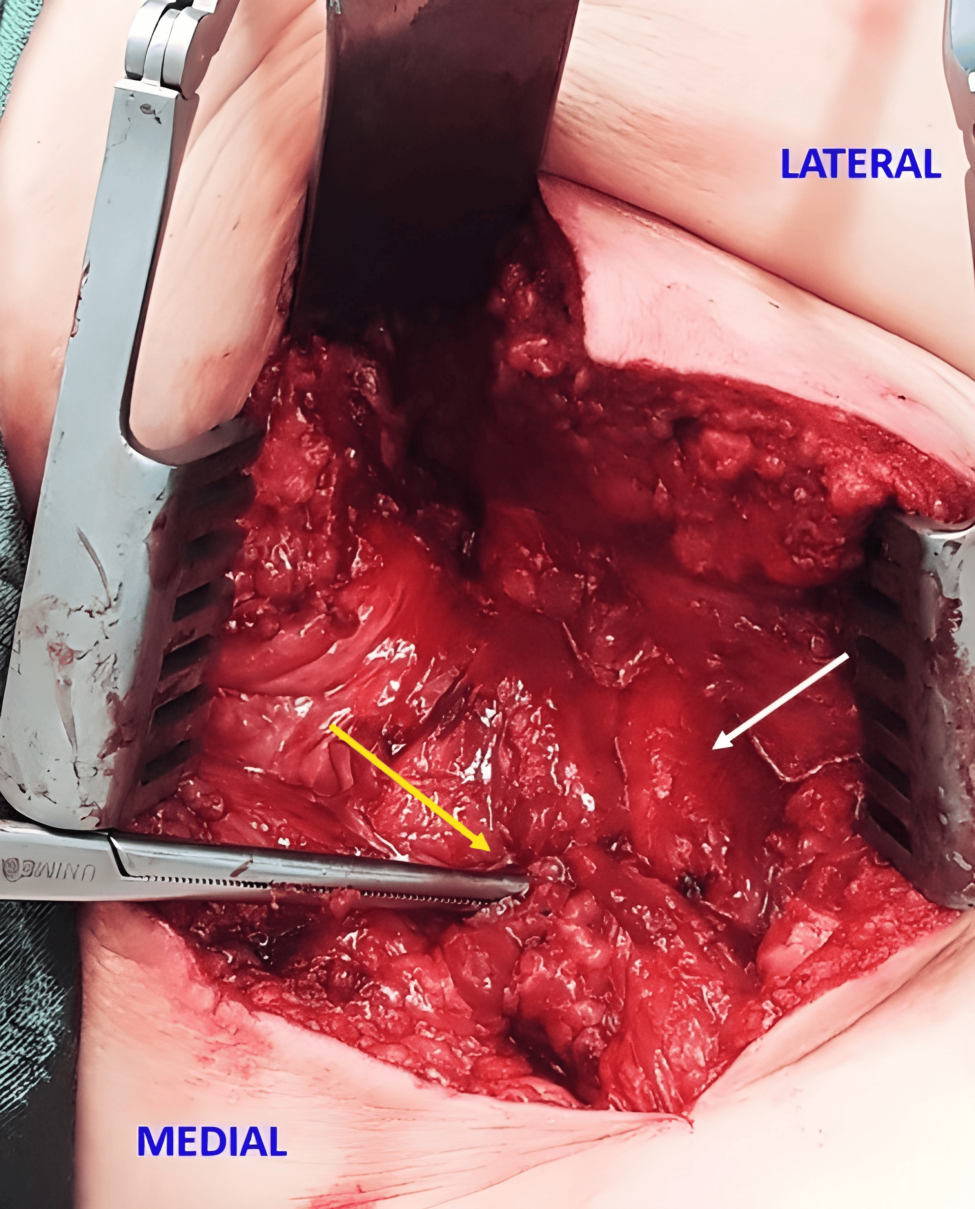
Intraoperative tendon stump and the sciatic nerve view Intraoperative view of the right thigh showing the tendon stump (yellow arrow) and the sciatic nerve (white arrow). Image Source: Faculty of Medical Science and Health, Pontifical Catholic University of São Paulo; Used with Permission

After epidural anesthesia, the patient is placed in the supine position with the table in reverse Trendelenburg to reduce tension on the sciatic nerve. A T-shaped skin incision is made, with a horizontal limb of approximately 7 cm over the gluteal fold and a vertical limb of 8 cm extending distally from its midpoint. The deep fascia is incised longitudinally with care to avoid injury to the posterior cutaneous nerve of the thigh. The hamstring tendon may be retracted and adherent to the surrounding soft tissues (Figures 1, 2). Great care is taken to avoid sciatic nerve injury, requiring meticulous dissection and protection, aided by 4x magnifying glasses.

**Figure 3:**
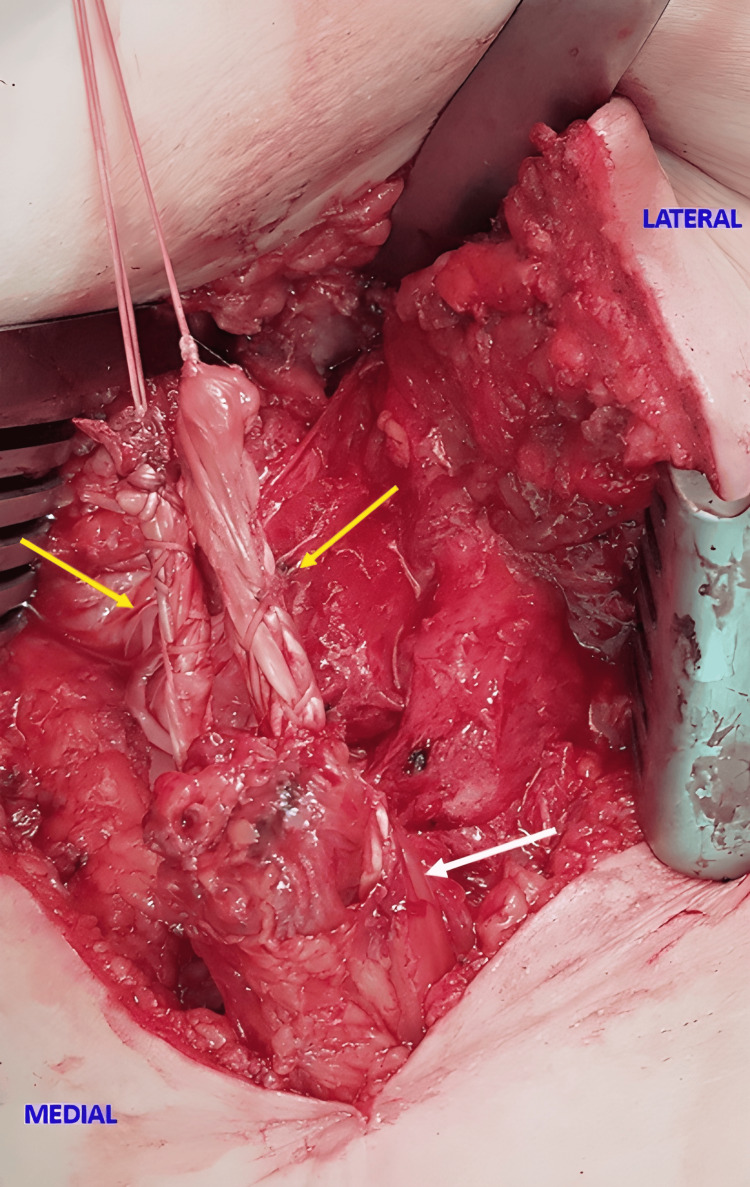
allograft arms passing through the tendon stump Intraoperative view of the right thigh showing the allograft arms (yellow arrows) passing through the tendon stump (white arrow). Image Source: Faculty of Medical Science and Health, Pontifical Catholic University of São Paulo; Used with Permission

While the hamstring stump is mobilized, a second team prepares a composite allograft by joining semitendinosus and gracilis tendons (about 20 cm each) with #2 Vicryl. The graft is then passed through the hamstring tendon stump from medial to lateral via a vertical incision made approximately 15 mm distal to the proximal end of the stump. After verifying that the medial and lateral portions of the graft are equal in size, two stitches are placed to secure the allograft to the hamstring tendon stump (Figure 3).

**Figure 4:**
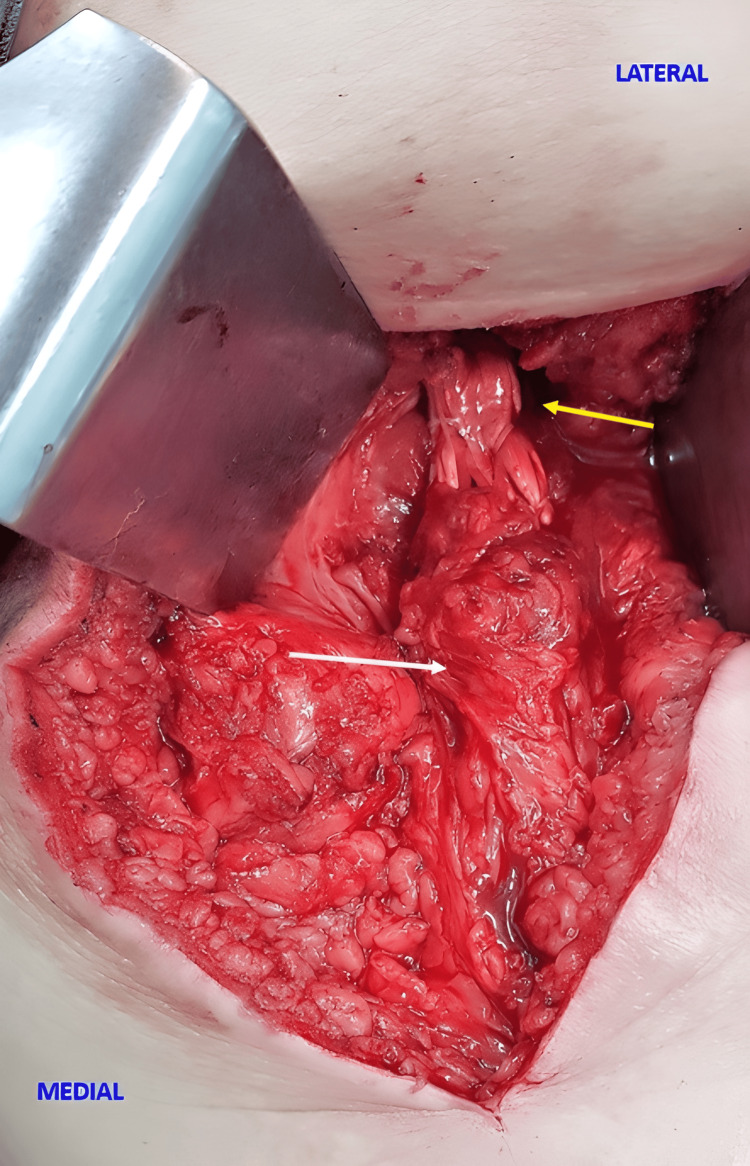
Intraoperative view of the allograft secured to the ischium and the tendon stump Intraoperative view of the right thigh showing the allograft secured to the ischium (yellow arrow) and the tendon stump (white arrow). Image Source: Faculty of Medical Science and Health, Pontifical Catholic University of São Paulo; Used with Permission

The ischial tuberosity is prepared for anatomic graft fixation using two 4.75 mm SwiveLock anchors. One graft end is attached to the medial origin of the conjoint tendon (semitendinosus and biceps femoris), and the other to the lateral origin of the semimembranosus, with a 15 mm gap between anchor points. The graft ends are then sutured together, forming a quadruple graft. After fixation (Figure 4), layered closure and elastic bandaging are performed.

**Figure 5:**
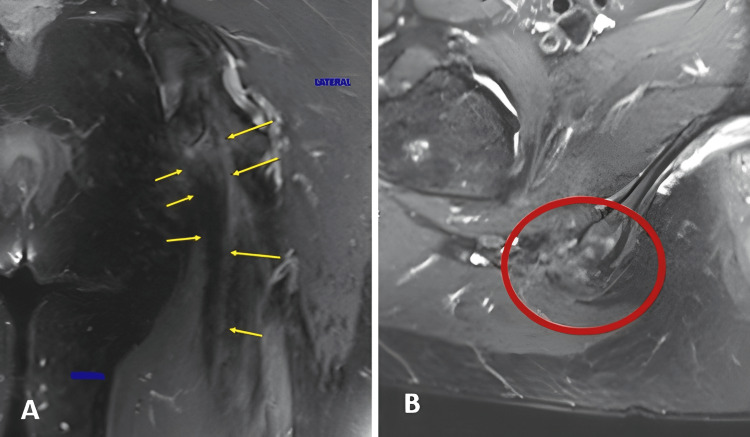
Postoperative MRI (A) Postoperative coronal T2 MRI showing the reinserted hamstring tendons at the ischial tuberosity (yellow arrows); (B) Postoperative axial T2 fat-suppressed image demonstrating the hamstring allograft reinsertion (red circle). Image Source: Faculty of Medical Science and Health, Pontifical Catholic University of São Paulo; Used with Permission

MRI confirming graft incorporation is shown in Figure 5.

Clinical pearls and pitfalls are summarized in Table 1.

**Table 1:** Clinical pearls and pitfalls of the surgical management of chronic proximal hamstring avulsion using hamstring allograft reconstruction

Category	Pearls (Key Tips)	Pitfalls (Common Risks)
Patient Positioning	Supine position with reverse Trendelenburg reduces sciatic nerve tension	Inadequate positioning may increase nerve traction and risk of neuropraxia
Incision Planning	A T-shaped incision centered over the gluteal fold provides adequate exposure while minimizing unnecessary dissection	Misplaced incisions may impair visualization and comprimise cosmesis
Nerve Protection	Careful dissection of the sciatic nerve in chronic cases with scarring is critical Use of 4× magnification glasses significantly improves visualization and safety	Dense scar tissue may obscure the nerve, increasing the risk of inadvertent damage
Graft Type	Soft tissue graft allows for two anchors placed 15 mm apart, avoids large tunnel drilling, reducing the risk of ischial tuberosity weakening	Slower biologic incorporation compared to bone plugs grafts
Rehabilitation	A custom-made brace maintaining the hip at 0° extension and the knee at 90° flexion protects the repair during early healing	The effectiveness of postoperative protocols relies on strict adherence to brace use and weight-bearing limits
Evidence Base	Based on multiple case series and anatomic logic	Lack of comparative biomechanical data for different graft / fixation strategies

Rehabilitation

**Figure 6:**
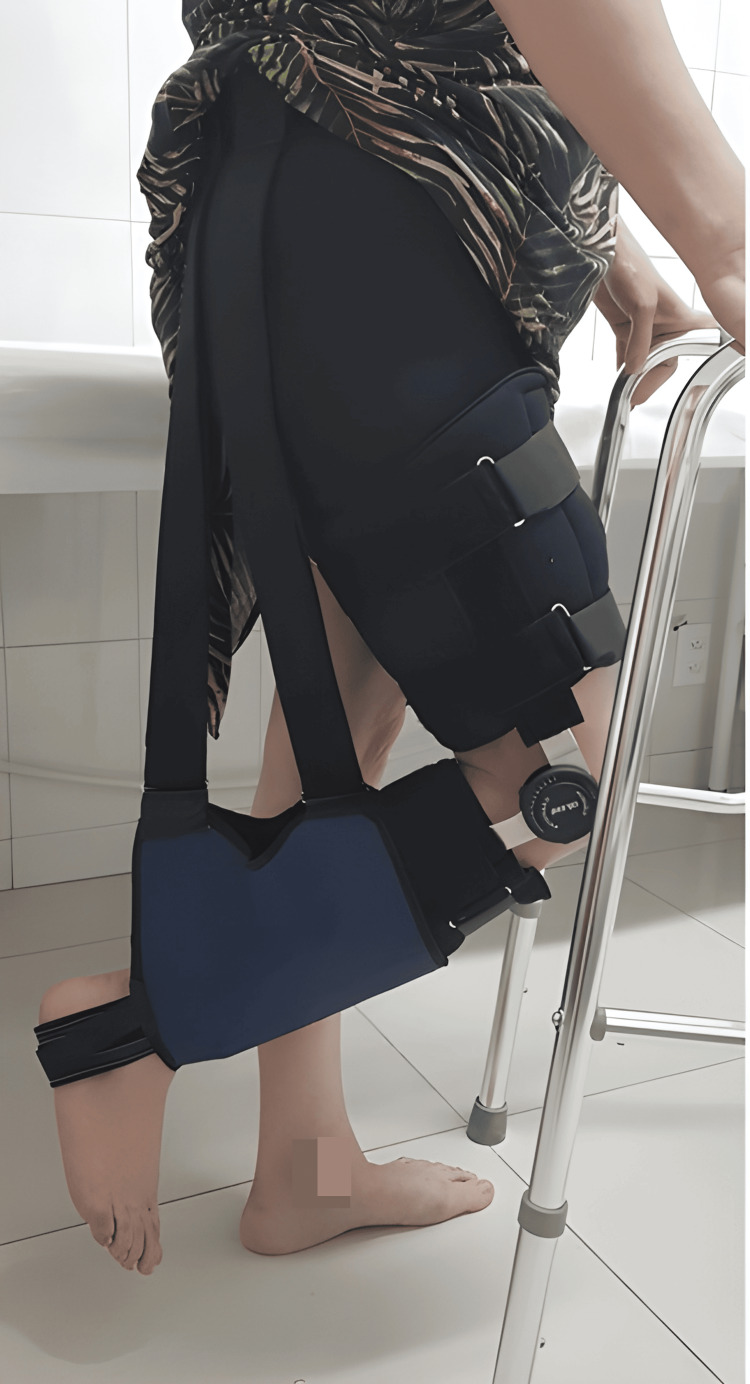
Postoperative custom-made brace used to immobilize the hip in 0° of extension and the knee in 90° of flexion. Image Source: Faculty of Medical Science and Health, Pontifical Catholic University of São Paulo; Used with Permission

Postoperatively, a custom-made brace is applied in the operating room and maintained for four weeks. It immobilizes the hip in 0° of extension and the knee in 90° of flexion [14], being worn day and night to prevent hip flexion and knee extension, thereby protecting the allograft fixation site (Figure 6). The elastic bandage is used during the first two weeks, and the sutures are removed at that time.

Patients use crutches for two to three weeks with light-touch weight-bearing, gradually progressing to full weight-bearing thereafter. Sitting was avoided for the initial two weeks. Light pool activities, including swimming, are introduced at four weeks, and cycling at four to six weeks, along with the initiation of isometric strengthening exercises. Hamstring stretching is restricted during the first four weeks. Range of motion exercises and weight-bearing are formally initiated at four weeks, with progressive strengthening exercises beginning at three months postoperatively [11,12,15].

## Discussion

Allografts or autografts are used to reconstruct significant defects in retracted proximal hamstring avulsions, aiming to restore anatomic continuity. While the Achilles tendon is the most commonly used allograft [7,15-17], other options include the ipsilateral distal iliotibial tract [12] and distal hamstring tendons [18]. No biomechanical comparisons among these grafts are available. We created a comparison table of the grafts described in the literature for the reconstruction of chronic hamstring avulsions with retraction (Table 2).

**Table 2:** Comparative analysis of graft options for chronic proximal hamstring avulsion

Graft	Typical Indication	Advantages	Disadvantages / Risks	Most Common Fixation
Achilles – allograft (with or without bone block)	Large defects with significant retraction; need for robust bridging; cases with sciatic neurolysis	Favorable length and caliber; avoids donor-site morbidity; good handling	Cost and availability; theoretical allograft risks	Anchors (knotted or knotless) on the ischium; sometimes transosseous tunnels
Semitendinosus ± gracilis – autograft (ipsilateral or contralateral)	Moderate defects; when allograft is to be avoided	Autologous biology; wide availability	Donor-site morbidity; may not reach very long defects	Graft sutured to stump and reinserted into ischium with anchors
Fascia lata – autograft	Autologous alternative “bridge” when distal tendons are not viable	Wide sheet for reinforcement	Variable strength and handling; lateral thigh morbidity	Sutured to stump and reinserted with anchors
Quadriceps – allograft	Recent option for extensive defects	Large caliber; adequate length	Same considerations of allograft; still limited experience	Fixation with multiple anchors
Reconstructions with distal hamstring tendons (ipsilateral autograft)	When distal hamstring tendons can be harvested for bridging	Avoids allograft; maintains the same muscle group (“family”)	May reduce residual strength; limited reach in long retractions	Anchors and/or tunnels

A hamstring allograft was chosen for its cylindrical shape (9-10 mm diameter), allowing anatomical reconstruction by anchoring to the medial origin of the conjoint tendon and the lateral origin of the semimembranosus. Folsom et al. reported the use of an Achilles tendon allograft with a bone plug, which was secured within a tunnel in the ischial tuberosity using a 9 × 25 mm metallic interference screw [17]. Similarly, Larson [7] and Rust et al. [15] used Achilles allografts with the bone plug fixed using a 7 or 8 × 20 mm interference screw. On the other hand, Saltzman et al. used an Achilles tendon allograft in a sandwich configuration, without a bone plug, to fill significant defects in cases of retracted proximal hamstring avulsion [16].

Sarimo et al. published a technique using an autologous free graft harvested from the ipsilateral distal iliotibial tract, fixed with suture anchors, to bridge the defect between the retracted hamstring tendons and the ischial tuberosity [12], and Ebert et al. reported the use of ipsilateral distal hamstring tendons to restore continuity in cases of proximal hamstring avulsion [18]. In their technique, the gracilis was passed in a ventral-to-dorsal direction, and the semitendinosus was passed from medial to lateral, creating a 90-90 configuration. The proximal ends of the tendons were whip-stitched, and multiple interrupted absorbable sutures were used to unify the grafts into a quadruple hamstring neo-tendon. Two suture anchors, placed 1 cm apart on the lateral ischial footprint, were used for graft fixation.

As can be observed, authors who used soft tissue grafts for fixation to the ischial tuberosity preferred anchors, whereas those who used bone grafts opted for interference screws measuring 7-9 mm [7,15,17]. However, considering the average dimensions of the ischial tuberosity (29.7 × 37.8 mm) [19], drilling a tunnel of 8-9 mm may compromise its integrity, while using a soft tissue graft with two anchors spaced 15 mm apart may represent a safer alternative.

We utilized a custom-made brace to immobilize the hip in 0° of extension and the knee in 90° of flexion for four weeks, as chronic avulsions often present with substantial retraction of the musculotendinous units [3]. Our postoperative protocol was based on previously described approaches [11,12,15]. Brace protocols vary among authors. Sarimo et al. did not advocate postoperative use of casts or orthoses [12]. Folsom et al. used a hinged knee brace set at 60-90° of flexion, gradually extended to full, over four to six weeks [17]. Larson recommended brace use for a total of six weeks, starting with the knee locked at 90° of flexion, progressively decreasing the flexion angle every two weeks [7]. Rust et al. applied a hinged knee brace with the knee locked at 90° of flexion, which was gradually extended to full extension over four to six weeks postoperatively [15]. Saltzman et al. placed patients in a hinged knee brace locked at 45° of flexion [16]. In cases of excessive tension on the reconstruction, a hip-knee orthosis was used, positioning the hip in neutral and the knee at 90° of flexion. Bracing was maintained for approximately six to eight weeks. Cain et al. used a knee brace locked at 90° for a total of six weeks [11], and Ebert et al. kept patients in a brace locked at 30° of flexion for two weeks postoperatively [18].

Our technique has some limitations, such as a lack of long-term follow-up data, slower incorporation, allograft availability, potential immune reactions, and variability in rehabilitation protocols.

## Conclusions

Chronic proximal hamstring avulsion can be surgically repaired using a hamstring allograft. This procedure allows for a more anatomic reconstruction, enhances functional recovery, improves persistent weakness, and provides significant pain relief. However, although this approach is promising, it requires further validation through larger studies with standardized outcome measures.
